# Artificial intelligence in anesthesiology education: transformative applications, challenges, and future perspectives

**DOI:** 10.3389/fmed.2026.1817855

**Published:** 2026-04-28

**Authors:** Cheng Chen, Shujing Xie, Zhihui Luo, Ziyan Hu, Xiaohong Du, Dan Huang, Haijun Hu, Shengliang Peng

**Affiliations:** 1 Department of Anesthesiology, the Second Affiliated Hospital, Jiangxi Medical College, Nanchang University, Nanchang, Jiangxi Province, China; 2 Key Laboratory of Anesthesiology of Jiangxi Province, Nanchang City, Jiangxi Province, China; 3 University Queen Mary School, Nanchang, Jiangxi, China; 4 The Second Clinical Medical College of Nanchang University, Nanchang, Jiangxi, China

**Keywords:** anesthesiology education, artificial intelligence, immersive learning, objective competency assessment, precision medical education

## Abstract

Artificial intelligence offers the potential to revolutionize anesthesiology education by enabling precision education, a data-driven approach to tailor learning experiences to individual needs, thereby moving beyond the constraints of traditional pedagogical methods. This review examines the emerging applications and potential impact of AI-driven technologies, from virtual reality simulators that facilitate deliberate practice of complex procedures to machine learning platforms that enable precision education and objective competency assessment. We highlight how these tools enhance procedural fluency, clinical reasoning, and educational management. Nevertheless, this technological advancement is accompanied by profound challenges, including the risks of de-skilling, the perpetuation of algorithmic biases, data security vulnerabilities, and issues of equitable access. We argue that AI’s role is as an augmentative tool, empowering educators to provide more personalized feedback and facilitate higher-order skill development, while also raising crucial ethical considerations. Navigating the future of anesthesiology education requires a balanced approach: embracing the benefits of AI while implementing robust governance to mitigate its risks, thereby fostering a new generation of anesthesiologists equipped to leverage technology for superior patient care. To this end, future research should prioritize rigorous validation of AI tools in clinical settings and focus on ethical guidelines for responsible AI implementation.

## 1 Introduction

Anesthesiology education encompasses standardized training for medical students, residents, and junior anesthesiologists, and is sustained throughout the entire career of anesthesiologists through continuing medical education. Traditionally, the basis of knowledge and skill transfer in this discipline has been pedagogical methodologies and mentorship, such as case discussions, seminars, lecture-based learning, and simulation training. While these methods are valuable, they possess inherent limitations. Didactic lectures, for instance, often foster passive learning, leading to a lack of student engagement and diminished motivation (1). A further potential challenge is that such lectures can demand significant development time from educators and, when conducted live, may necessitate scheduling accommodations to free faculty from clinical responsibilities. Similarly, conventional simulation training may fail to provide a fully immersive experience, leaving trainees mentally unprepared for the pressures and complexities of real-world clinical scenarios. These limitations have prompted educators and researchers to seek innovative pedagogical strategies. In this context, Artificial Intelligence (AI), including machine learning (ML) and deep learning (DL), is emerging as a potential tool for precision education in anesthesiology, opening up entirely new possibilities for enhancing learning and assessment outcomes (2).

In a broad sense, AI refers to machines that analyze and learn from vast amounts of data using algorithms to efficiently solve complex problems (3), encompassing sub-fields such as ML and DL. ML is a popular branch of artificial intelligence that employs various techniques to learn from patterns in real-world datasets and predict future outcomes, thereby solving complex problems involving big data. DL simulates the human brain’s operation through multi-layered artificial neural networks, which can automatically generate predictions from input (training datasets) (3). Its application is rapidly expanding within clinical practice, and medical education is similarly experiencing the emergence of innovative AI-driven educational methodologies. For example, generative conversational AI can simulate virtual patient interactions, offering tailored training for clinical trainees (4). Furthermore, AI-driven systems are being developed to automate the assessment of trainee performance in simulated environments. The field of anesthesiology is also actively exploring these advancements. For instance, one study developed a ML model using audio-visual, speech transcription, and vital sign data from high-fidelity simulation videos of intraoperative anaphylaxis to automatically evaluate the performance of anesthesiology trainees in simulated intraoperative anaphylaxis scenarios (5). A growing body of evidence underscores the positive impact of AI on enhancing pedagogical methods and educational outcomes. Although the use of AI in the medical field is increasing, AI education lags far behind. Data shows that nearly 40% of medical students actively use AI tools like ChatGPT, yet only about 5% have received formal training in their use. Furthermore, only approximately 4% have received education on AI ethics. This “disconnect between usage and education” highlights the urgent need for formal integration strategies to prevent uncritical reliance on these technologies (6).

Leveraging this potential, AI is anticipated to assume a progressively integral role in anesthesiology training paradigms. This review aims to synthesize recent research on the application and impact of AI in this specific educational domain. We will investigate how AI technologies are transforming teaching methodologies, enabling objective assessment, and improving educational management. Subsequently, we critically analyze the accompanying challenges and ethical complexities. Finally, we examine prospective pathways for the integration of AI into anesthesiology education and training. By synthesizing this body of knowledge, this review seeks to inform educators and researchers on leveraging AI’s benefits while mitigating its risks, ultimately contributing to the cultivation of a new generation of highly skilled anesthesiologists.

## 2 Methods

This review synthesizes the existing literature on the applications of AI in anesthesiology education. Given the broad and rapidly evolving nature of this interdisciplinary field, a structured narrative review approach was adopted to provide a comprehensive overview while maintaining methodological transparency.

### 2.1 Search strategy

A structured literature search was conducted in two stages: an initial search in June 2024 and a final updated search in March 2026. The following electronic databases were searched: PubMed/MEDLINE, Web of Science, and the Education Resources Information Center. The databases were selected to cover three complementary domains: PubMed/MEDLINE for biomedical and clinical literature, Web of Science for interdisciplinary and high-impact research, and ERIC for education-specific scholarship. This combination aimed to capture both the clinical anesthesiology literature and the educational methodology literature relevant to the review scope. The search strategy used keywords related to three core concepts: (1) Artificial Intelligence (e.g., “artificial intelligence,” “machine learning,” “deep learning,” “generative AI,” “large language model,” “virtual reality,” “adaptive learning”); (2) Anesthesiology (e.g., “anesthesiology,” “anesthesia,” “anaesthesia,” “regional anesthesia,” “regional anaesthesia,” “nerve block”); and (3) Education (e.g., “medical education,” “anesthesia education,” “anaesthesia education,” “anesthesiology training,” “simulation training,” “clinical skill,” “teaching,” “learning”). Within each theme, synonymous terms were combined using the Boolean operator “OR” and the three themes were then combined using “AND.”

To enhance search sensitivity and account for terminological variation in the literature, we considered a broader set of synonymous terms during the study selection phase, including “neural network,” “natural language processing,” “augmented reality,” “intelligent tutoring system,” “competency-based assessment,” “perioperative,” “airway management,” “pain management,” “ultrasound-guided regional anesthesia,” “curriculum,” “assessment,” and “feedback.” Given the broad and heterogeneous nature of the literature, we did not apply these expanded terms as formal Boolean search strings, so as to maintain a manageable retrieval volume for screening, consistent with the narrative review approach. Instead, we identified relevant studies through manual screening of search results, cross-referencing bibliographies of included articles, and consultation with content experts.

**Figure 1:**
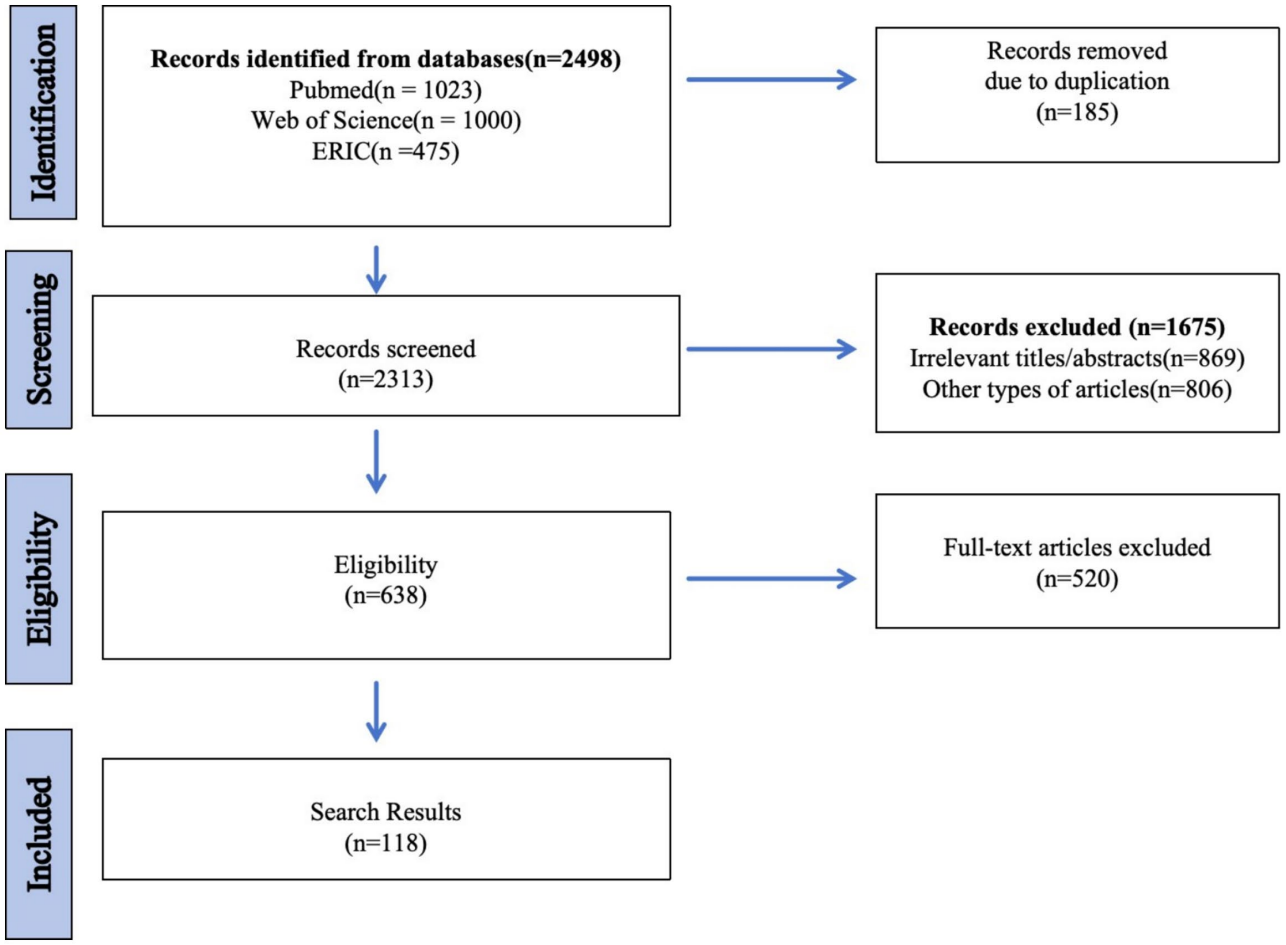
Literature search and study selection process.2.2. Inclusion and exclusion criteria.

Based on the core search strategy, Figure 1 summarizes the literature screening process. Given the narrative scope of this review, the search aimed for broad coverage of representative and seminal literature rather than exhaustive retrieval.

Inclusion criteria required that articles: (1) were published in peer-reviewed journals or as preprints and written in English; (2) focused on AI applications in anesthesiology education or training; (3) involved medical students, residents, fellows, or practicing anesthesiologists as learners; and (4) were original research, reviews, or conference proceedings presenting empirical data or robust theoretical frameworks.

Studies were excluded if they: (1) focused on clinical AI applications without an educational component; (2) discussed AI in non-anesthesiology medical fields; or (3) were unavailable in full text.

### 2.2 Study selection and data synthesis

Two reviewers initially screened titles and abstracts to identify potentially relevant studies. They then retrieved and assessed the full texts of these articles against the inclusion criteria. Any disagreements were resolved through discussion or by consulting a third reviewer. Given the heterogeneity of the included studies, ranging from randomized controlled trials to qualitative surveys, and the broad scope of this review, we conducted a narrative synthesis. We extracted key findings and grouped them thematically into the following categories: immersive training, personalized learning, competency assessment, and educational management. Due to the broad scope and heterogeneous nature of the included studies, we did not perform a formal quality assessment; instead, we prioritized transparency in study selection to minimize bias.

### 2.3 Evidence level classification

To provide readers with a transparent assessment of the evidence strength underlying the applications discussed in this review, we classified the included studies according to their study design. We assigned evidence levels using a framework adapted from the Oxford Centre for Evidence-Based Medicine (OCEBM) 2011 levels of evidence, modified for applicability to educational research contexts where randomized controlled trials are not always feasible or ethically appropriate (7).

### 2.4 Quality appraisal

To ensure the methodological quality of this narrative review, we assessed the manuscript using the Scale for the Assessment of Narrative Review Articles (SANRA). SANRA is a six-item instrument developed specifically for evaluating non-systematic reviews, covering: (1) explanation of the review’s importance, (2) statement of the aims, (3) description of the literature search, (4) referencing quality, (5) scientific reasoning, and (6) appropriate presentation of data. Each item is scored from 0 (low standard) to 2 (high standard), with a maximum total score of 12 (8).

## 3 The application of major AI technologies in clinical anesthesia

The integration of AI into anesthesiology education is driven by its expanding clinical applications. AI has developed from a novel concept into a clinically valuable tool. ML, a core subset of AI, and its branch DL, show good application prospects in supporting medical care throughout the perioperative period. Preoperatively, although ML algorithms can generate anesthesia plans by analyzing patient data (9), there is a lack of primary research evidence indicating that ML-driven anesthesia plans are accurate and beneficial. Therefore, it remains necessary for anesthesiologists and multidisciplinary teams to make clinical decisions collaboratively (10). However, studies have shown that existing ML models can help anesthesiologists develop personalized and effective anesthesia strategies and enhance patient safety. For example, Te et al. (88) used structured data containing features to build different types of ML models to predict post-intubation hemodynamic instability indices, which can indicate the potential changes in a patient’s hemodynamic status during surgery. At the same time, based on the varying importance of features in the models, anesthesiologists can prioritize the factors with higher significance before surgery and adjust certain controllable features, such as the initial dosage of administered drugs, to assess and prevent potential risks (11). This was a large-sample, single-center, retrospective study without external validation, real-world clinical trials, or patient-important outcome assessments. Furthermore, it only included patients with American Society of Anesthesiologists Physical Status (ASA-PS) class I–II, indicating limited generalizability to high-risk populations. Further research is needed to investigate the actual clinical value of this ML model. Furthermore, Yoon et al. developed a Natural Language Processing (NLP)-based ML model that can classify ASA-PS levels using free-text preanesthetic evaluation summaries. Compared with anesthesiologists, the ClinicalBigBird model demonstrated higher specificity, accuracy, and F1-score than board-certified anesthesiologists. This model enables automated and relatively stable classification of preanesthetic evaluation summaries with accuracy comparable to certified anesthesiologists, which may theoretically help streamline the preoperative risk assessment process and provide an objective basis for personalized anesthetic management (12). However, this study was a single-center retrospective investigation conducted in adult patients at a hospital in South Korea, resulting in a relatively homogeneous study population and limited clinical settings. Furthermore, the ClinicalBigBird model was trained and validated on preanesthetic evaluation texts prepared by anesthesiologists, rather than complex, real-world raw clinical data such as original electronic medical records. Notably, the model was not implemented into actual clinical workflows, and no impact on patient clinical outcomes was assessed. Nevertheless, the above findings still suggest that ML holds potential value in assisting anesthesiologists in effective preoperative anesthesia risk assessment.

Intraoperatively, ML models are being explored for the monitoring of adverse events. For example, a Randomized Controlled Trial (RCT) preliminarily evaluated an ML-derived early warning system for hypotension in a real clinical setting. The results showed that, compared with standard care, the use of this ML warning system could reduce the time-weighted average of hypotension during surgery and provide anesthesiologists with early warnings and real-time objective decision support (13). However, this study was a single-center, small-sample RCT, did not assess patient clinical outcomes, and involved patients with relatively severe conditions and a higher risk of hypotension, which may exaggerate the warning effect. The study was unblinded, and anesthesiologists using the system may have been more alert to hypotension, introducing a risk of bias. This system is still in the clinical exploration stage, and its safety and generalizability need to be validated in diverse clinical settings. And AI based on ML and DL has shown promising potential in ultrasound imaging for regional anesthesia. For instance, the ScanNav Anatomy Peripheral Nerve Block device utilizes DL based on Convolutional Neural Networks, applied in the field of computer vision, to predict and overlay colors on real-time ultrasound images, highlighting relevant anatomical structures. An external validation study superimposed color markers from the ScanNav device onto original ultrasound scan videos obtained without the device, and experts compared and evaluated the original videos against the overlaid versions. According to expert evaluation, the mean accuracy of color overlay on key anatomical structures was 93.5%, and this highlighting function may help reduce the risk of puncture injury and nerve block failure (14). However, the study conclusions were based on retrospective video analysis and expert subjective judgment, with no evidence from real-patient clinical scenarios or patient outcomes. The actual clinical benefits of the anatomical structure highlighting the function of the ScanNav device remain to be verified in prospective clinical trials. Similarly, Bowness et al. (14) conducted a prospective, randomized exploratory study and reported that operators using the DL-based ScanNav device achieved higher accuracy in obtaining standard block views and identifying ultrasound anatomical structures than those without device assistance when performing ultrasound scanning on healthy volunteers (15). However, this study had several limitations. First, the sample size was relatively small. Second, the evaluating experts were aware of device usage, and no blinding was applied, which could introduce information bias and potentially overestimate the supportive effect of the device. Third, ultrasound scanning was performed on healthy volunteers rather than in real complex clinical surgical settings. In addition, only operation-level indicators were assessed, with no evaluation of clinical outcomes such as puncture complications or block success rates. Therefore, the clinical applicability of this device remains to be confirmed. Besides, Kowa et al. (15) performed a prospective randomized study with junior anesthetists and found that the DL-based ScanNav device, which generates color overlays for sonoanatomical structures, sustained improvements in Ultrasound-guided Regional Anesthesia (UGRA) scanning performance, including appropriate block view acquisition and participant confidence, even at a 2-month follow-up. This study demonstrated that the DL-based ScanNav device provides sustained intraoperative procedural assistance in simulated settings. However, it only validates tool performance and does not assess genuine educational benefit, improvement in operators’ independent performance, or real patient and clinical outcome measures.

Postoperatively, DL contributes to managing anesthesia emergence and predicting patient care needs (16). For instance, Fontaine et al. found that a DL system based on Convolutional Neural Networks, utilizing facial expression analysis, can distinguish facial expressions associated with different pain intensities, predict pain scores, and demonstrate high sensitivity in diagnosing moderate and severe pain. This DL algorithm may provide some objective reference for physicians to adjust analgesic regimens (17). However, this was a single-center performance validation study. The developed DL system predicted pain scores by analyzing facial images. Although the overall sample size was relatively large, the external validation set included only 120 facial images, which may compromise the reliability of the results. While the system showed high sensitivity for moderate to severe pain, the high proportion of pain-free facial images (44%) could lead to considerable underdiagnosis in real clinical scenarios with high pain prevalence. In addition, the pain scores used in the study were somewhat subjective, and the analyzed facial images were static rather than allowing continuous pain monitoring. Furthermore, the study did not investigate the effect of clinical interventions or evaluate the impact on patient pain management or clinical outcomes. Despite these limitations, the study preliminarily supports the potential of deep learning in postoperative pain assessment and may provide auxiliary support for the management of anesthesia emergence. Crucially, as ML and DL serve assistive roles, anesthesiologists should engage in continuous professional development to effectively leverage these technologies and evolve their expertise (18).

## 4 The application of AI in anesthesia education

The same algorithms that power these clinical applications provide insights for the development of next-generation educational tools, which we will explore in the following sections.

### 4.1 AI-assisted clinical skills and simulation training

AI can provide intelligent support for skills training and simulation-based education in anesthesiology through its capabilities in image recognition and real-time data processing. For example, a simulation-based study using mannequins and a physical nerve block simulator indicated that, compared with traditional training, an AI-assisted identification system enables residents to better master ultrasound-guided popliteal sciatic nerve block. During the first month of clinical practice, the AI training group exhibited a significantly lower incidence of patient-reported paresthesia and scored higher on both objective assessment scales and self-evaluation (19). Moreover, the study only enrolled 40 anesthesiology residents from a single hospital, representing a single-center, small-sample simulation-based teaching study. No clinical bedside teaching was performed in either group; only one-month post-training performance was evaluated, with no data on long-term skill retention. Additionally, the relatively simple popliteal sciatic nerve block was selected, introducing potential risk of bias. Although the findings suggest that this AI-assisted nerve identification system may improve simulation training effectiveness and short-term clinical performance among anesthesiology residents, the generalizability and clinical applicability of the conclusions are limited.

As an independent simulation modality, virtual reality (VR) can create highly realistic and repeatable immersive virtual scenarios, providing a low-risk training environment that supports repeated practice in anesthesia education. For instance, Zheng et al. developed a virtual anesthesia platform combining VR technology and haptic feedback. A questionnaire survey showed that 70% of anesthesia interns agreed that the platform exhibited high repeatability, improved anatomical recognition, and provided a distinct sense of breakthrough during puncture procedures. The platform not only delivers a highly immersive experience to enhance procedural realism, but also, more importantly, enables the collection of objectively quantifiable performance data such as motion trajectory, hand-eye coordination, and error rates (20). These data are expected to serve as fundamental input for the objective, data-driven assessment systems discussed in Section 4.3, thereby establishing a closed-loop educational ecosystem. Notably, VR is not part of AI; the two technologies are independent of each other, but AI can help improve the training effectiveness of VR simulations through its capabilities for real-time feedback and intelligent assessment. While previous reviews highlighted the potential of AI, recent randomized clinical trials provide robust evidence of its efficacy. Fazlollahi et al. demonstrated that an AI tutoring system (Virtual Operative Assistant) resulted in significantly higher expertise scores and better skill transfer in simulated neurosurgical tasks compared to remote expert instruction. Notably, the AI tutor achieved these superior technical outcomes without increasing learners’ cognitive load or negative emotions, suggesting that AI can effectively augment human mentorship in procedural training (21). The study only enrolled 70 medical students, with a small sample size and limited statistical power. Furthermore, the recruited volunteers may have been relatively more motivated, introducing selection bias. A study has shown that the sepsis care performance of nursing students trained through AI-driven VR simulations did not differ significantly from that of students trained via human-controlled VR simulations; however, medical students in the AI-driven group demonstrated a significant improvement in sepsis care knowledge compared to their baseline (22). Although the two aforementioned studies derive from the fields of surgery and nursing, their results consistently demonstrate that VR provides medical students with a low-risk, repeatable simulated training environment, while AI has positive potential in improving the effectiveness of skills training and professional knowledge. The combination of AI and VR can also provide a new direction for clinical skills teaching in anesthesiology. A Spanish publication described a training module for residents and anesthesiologists developed within an AI- and VR-based learning simulation system. In this system, VR constructs the immersive virtual environment, presents virtual anatomical models and clinical case scenarios, and collects procedural performance data. AI algorithms are used to enable personalized learning interaction, analyze operational data, and provide targeted feedback, thereby helping to enhance the teaching of pain management and UGRA (23). However, this publication mainly focused on the introduction of technical applications, provided no objective data on effectiveness, and did not validate the transfer of trained skills to clinical practice. Also, we have provided a table that summarizes the key points of the above research.

### 4.2 Personalized cognitive development: adaptive learning and knowledge platforms

Beyond psychomotor skills, AI is revolutionizing cognitive development in anesthesiology education by enabling adaptive and personalized learning experiences. AI-driven platforms leverage data analytics to tailor educational content, address individual knowledge gaps, and optimize the acquisition of clinical reasoning skills.

A key manifestation of this is the Intelligent Tutoring Systems (ITS), an AI-powered platform that integrates extensive educational resources and adapts to individual learner needs. ITS can identify areas where students face learning difficulties or need improvement by interacting with learners and assessing their performance, and provide personalized recommendations. For example, Furlan et al. (24) developed a virtual patient simulator that integrates NLP and an ITS, allowing students to engage in clinical diagnostic reasoning with the virtual patient. This virtual patient simulator can provide real-time feedback, offering students specific knowledge areas that need review and helping them target and fill gaps in their understanding. Short-term learning test results indicated that undergraduates improved their mastery of clinical knowledge related to the simulated cases after using the simulator. However, the study employed a single-group pre-post design without a parallel control, resulting in a high risk of bias. Only 15 undergraduate students were included, representing a small sample size with a single assessment outcome. Moreover, only short-term improvements in academic performance were observed, which cannot confirm the sustained educational value of the virtual patient simulator. Further large-sample, controlled studies are still required for validation. In addition, a novel AI navigation system utilizing augmented reality provides learners with specific reference points by displaying overlaid color labels and pulmonary tree diagrams, while delivering real-time feedback and evaluation during bronchoscopy procedures. Results from the randomized controlled trial indicated that intensive care physicians trained with the AI system perform bronchoscopy more quickly compared to those guided by professional human instructors (25). However, the study has several limitations. As a single-center, small-sample simulation-based teaching study using augmented reality, its generalizability of evidence is relatively limited. Furthermore, the study was conducted only in a virtual simulation environment without verifying skill transfer, and there is a risk of bias due to the non-blinded design. In addition, only short-term procedural performance was evaluated, with no evidence of long-term skill retention. Therefore, although the AI-based training model for bronchoscopy shows promising application potential, its value in anesthesiology education still needs to be confirmed by more targeted studies.

Moving beyond the delivery of structured knowledge, generative AI and NLP techniques further personalize the cultivation of higher-order cognitive skills, particularly in clinical reasoning. It can generate simulated virtual scenarios that augment traditional online video demonstrations. Within these safe learning environments, students can engage in diagnostic and management decisions for virtual patients, thereby translating theoretical knowledge into practical cognitive skills and enhancing clinical judgment (26–29). For instance, the intelligent virtual case-based learning system based on NLP technology allows students to simulate the role of a physician, collect case information, and engage in analysis and consideration to make diagnostic and treatment decisions. The system provides feedback after the students’ learning, listing the correct diagnostic factors and hypotheses while addressing knowledge gaps. Research results indicated that this system can enhance the clinical thinking of medical students, including both undergraduates and postgraduates, with a particularly notable improvement in logical reasoning (30). Of note, this study was not conducted in the anesthesiology specialty. In addition, the single-group, small-sample, pre-post self-controlled design precluded establishing a causal relationship between AI-assisted teaching and improved logical reasoning ability, and evidence regarding clinical translation and long-term effects was lacking. Nevertheless, this study suggests that AI-assisted virtual simulation and personalized feedback hold potential value for enhancing clinical reasoning training and may provide a feasible direction for clinical reasoning teaching in the anesthesiology specialty (see Table 1).

**Table 1:** Summary of key information from five major included studies.

References	Population and sample size	AI intervention	Main results	Primary and secondary outcomes
Yoon et al. (2024) (12)	Surgical adult patients Total *n* = 717,389 Test set *n* = 460	ClinicalBigBird, BioClinicalBERT, GPT-4 Automated ASA-PS classification from preoperative free-text notes	1. ClinicalBigBird > Board-certified anesthesiologists: Specificity 0.901 > 0.897; Precision 0.732 > 0.715; F1 0.716 > 0.713 (all *p* < 0.01) 2. ClinicalBigBird > Anesthesiology residents: Sensitivity 0.723 > 0.598; Specificity 0.901 > 0.868; F1 0.716 > 0.633 (all *p* < 0.001) 3. Misclassification rate: Model 11.74% < GPT-4 11.95% < Physicians 13.48% < Residents 21.96%	1. Macro-average AUROC: ClinicalBigBird = 0.915 > BioClinicalBERT = 0.899 > GPT-4 = 0.893 2. Specificity: 0.901 > 0.897 > 0.868 3. Precision: 0.732 > 0.715 4. F1-score: 0.716 > 0.713 > 0.633
Wijnberge et al. (2020) (13)	Elective non-cardiac surgery patients with invasive BP monitoring Randomized *n* = 68, completed *n* = 60 Intervention *n* = 31, control *n* = 29	ML–derived hypotension early warning system (HPI) + hemodynamic management protocol	1. Intervention < Control: Time-weighted average hypotension 0.10 < 0.44 mmHg (*p* = 0.001) 2. Intervention < Control: Total hypotension duration 8.0 < 32.7 min (*p* < 0.001) 3. Intervention < Control: Treatment response time 53 < 87 s (*p* < 0.001) 4. Serious adverse events: 0 < 2 cases	Primary: Time-weighted average hypotension (0.10 vs. 0.44 mmHg) Secondary: Total hypotension time, response time, hypertension rate, adverse events
Bowness et al. (2023) (86)	Twenty-one non-expert anesthetists, 5 healthy volunteers Total scans *n* = 126 AI-assisted *n* = 63, unassisted *n* = 63	ScanNav™ AI real-time anatomical labeling for ultrasound-guided regional blocks	1. AI-assisted > Unassisted: Correct block view rate 90.3% > 75.1% (*p* = 0.031) 2. AI-assisted > Unassisted: Correct structure identification 88.8% > 77.4% (*p* = 0.002) 3. Scan time, confidence, global score: No difference between groups	Primary: Correct block view rate (90.3% vs. 75.1%) Secondary: Structure identification (88.8% vs. 77.4%), confidence score, global rating, scan time
Fontaine et al. (2022) (17)	Postoperative patients Total patients *n* = 1,189; Images *n* = 2,810 External test set *n* = 120	ResNet-18 DL model Pain intensity (NRS 0–10) prediction from facial expressions	1. AI > Nurses: Pain classification accuracy 53% > 14.9% 2. AI < Nurses: Mean absolute error 2.4 < 3.04 3. AI > Nurses: Sensitivity for pain ≥4: 89.7% > 44.9% 4. AI > Nurses: Sensitivity for severe pain ≥7:77.5% > 17.0%	Primary: Classification accuracy (53% vs. 14.9%), mean absolute error (2.4 vs. 3.04) Secondary: Sensitivity, specificity, PPV, NPV for pain detection
Fazlollahi et al. (2022) (21)	Medical students (preclinical years 0–2) Total *n* = 70 AI tutoring *n* = 23, expert instruction *n* = 24, control *n* = 23	Virtual Operative Assistant (VOA) AI tutoring system VR brain tumor resection with quantitative audiovisual feedback	1. AI > Expert > Control: Practice expertise score improvement +0.66 > 0 > 0 (*p* < 0.001) 2. AI > Expert: Realistic simulation score higher by 0.53 points (*p* < 0.001) 3. AI > Control: Realistic simulation score higher by 0.49 points (*p* < 0.001) 4. Cognitive load and emotions: AI = Expert	Primary: ICEMS expertise score (+0.66 vs. 0 vs. 0) Realistic performance: AI = 0.14, Expert = −0.62, Control = −0.56 OSATS global score: 4.63 vs. 4.40 vs. 3.86(ns) Cognitive load: No difference among groups

AI, Artificial intelligence; DL, deep learning; ASA-PS, American society of anesthesiologists physical status; AUROC, area under the receiver operating characteristic curve; HPI, hypotension prediction INDEX; ICEMS, intelligent continuous expertise monitoring system; OSATS, objective structured assessment of technical skills; NRS, numeric rating scale; VR, virtual reality; VOA, virtual operative assistant; ML, machine learning; PPV, positive predictive value; NPV, negative predictive value.

Furthermore, AI platforms continuously track and analyze a learner’s interactions and performance data. This facilitates precision education, wherein the system provides individualized feedback and dynamically adapts the learning pathway based on each student’s progress and identified areas for improvement (21, 24, 31, 32). This data-driven approach facilitates interactive problem-solving and offers timely, personalized developmental guidance. For instance, an AI-assisted system (Virtual Operative Assistant) can classify medical students’ performance during simulated surgical skills training based on quantitative metrics (such as proficient or novice) and provide students with personalized analysis and specific behavioral improvement suggestions guided by operational indicators. Moreover, randomized clinical trial results have shown that in surgical simulation training, learners using the AI-assisted system achieve better skill acquisition compared to remote expert instruction (21). The study included 70 medical students with a relatively small sample size. As participants may have been relatively motivated, selection bias was present. Moreover, the research only involved virtual simulation for brain tumor resection, representing a single scenario with no clinical translation or long-term longitudinal follow-up data; thus, the durability of skill improvement could not be determined. In this study, the AI-assisted system was compared with remote expert teaching rather than face-to-face expert instruction. Although this research focused on surgical training, it confirmed the advantages of AI in quantitative assessment and personalized feedback, which can provide important references for skills training in anesthesiology, such as ultrasound-guided regional blockade and airway management. AI-based adaptive learning algorithms can dynamically adjust training strategies based on learners’ operational performance and proficiency, optimize learning paths, and help learners master professional knowledge and clinical skills. For example, Kumar et al. developed a VR platform integrated with AI-enhanced haptic feedback, through which trainees can receive medical skills training, including emergency response. VR constructed immersive virtual scenarios and visualized three-dimensional anatomy. The platform captured operational data and completed performance evaluation and scoring, while the AI algorithm dynamically adjusted scenario difficulty based on the scoring results to form an adaptive learning loop that gradually improved clinical skills. The study results indicated that this AI-driven adaptive feedback platform can reduce error rates by 45% and increase success rates by 42%, helping to improve the clinical performance of medical trainees. This improvement is not solely attributed to AI-based adaptive learning, but results from the combined effects of VR, haptic feedback, and adaptive algorithms. The specific role played by AI remains to be verified and clarified in future targeted studies. However, this study focused on general medical education without validation specifically in anesthesiology. Furthermore, all results were obtained in a simulated setting, and no real-world clinical translation was assessed (28).

### 4.3 Data-driven assessment and feedback: moving beyond subjectivity

AI is revolutionizing the assessment of clinical competence by introducing data-driven, objective methodologies that mitigate the inherent subjectivity of traditional evaluations. ML-based assessment tools, a subset of AI applications, can quantitatively evaluate narrative texts through automated scoring, simplifying performance tracking processes, providing real-time feedback, and conducting clinical skills assessments, particularly in psychomotor skills (32, 33). AI in anesthesiology can assess the depth of anesthesia, predict events and risks, evaluate ultrasound-guided procedures, and identify pain (34). By leveraging computational models such as deep learning and convolutional neural networks, AI can process diverse data inputs, including text and video, to generate robust and efficient performance analyses, thereby mitigating human bias and resource limitations (35, 36).

A key application is the automated assessment of procedural skills. AI is capable of analyzing video recordings of surgical procedures performed on patients for automated assessment. Specifically, ML can analyze data, such as operator video analysis, hand motion analysis, ultrasound video analysis, needle tracking, or eye-tracking technology, to assess the quality of an operator’s performance in ultrasound-guided regional anesthesia (9, 37). A systematic review indicated that ML methods such as hidden markov models, support vector machines, and artificial neural networks can analyze kinematic or visual data to assess surgical skills, including real surgeries, with an accuracy of over 80%, providing an evidence base for the future development of ML-based assessment tools that can offer clinically valuable feedback (38).

The integration of these multimodal data streams allows ML-based analytics systems to generate a holistic competency profile for each learner. This supports precision education through the delivery of timely, personalized, and actionable feedback. Furthermore, by analyzing trends in performance data, AI can predict future learning trajectories and identify areas requiring intervention, with the accuracy of these predictions being validated through training and testing datasets (31, 35, 39). For instance, AI can automatically analyze procedural performance data collected in virtual simulation training and provide trainees with personalized, objective feedback. Trainees can then conduct targeted improvements and practice based on this feedback to enhance their anesthesia skills (23). To mitigate potential biases inherent in AI-driven evaluations, a programmatic assessment approach is recommended. This approach should strategically combine insights from AI learning analytics with the nuanced judgment of human experts, such as faculty and clinical supervisors, to ensure a valid, reliable, and equitable evaluation of student performance and anesthesiology education effectiveness (31, 36, 40).

### 4.4 Enhancing educational management and resource optimization

AI’s impact extends beyond direct teaching and learning to significantly enhance the efficiency and precision of anesthesiology education, management, and resource allocation (Table 2). This optimization occurs at two primary levels: instructional intervention and institutional resource management.

**Table 2:** Key applications of artificial intelligence in anesthesiology education.

References	Application domain	Specific applications	Educational value	Representative examples	Evidence level
Zheng et al. (2023) (20)	Immersive psychomotor training	AI-powered simulators and VR platforms with haptic feedback for procedural practice.	Provides a safe, repeatable environment for deliberate practice; enhances procedural fluency and manual skill acquisition.	VR platform for combined spinal-epidural anesthesia with real-time performance tracking.	III (20)
Su et al. (2023) (26), Yunoki et al. (2018) (27), Turner et al. (2024) (35), Ara Shaikh et al. (2022) (87)	Personalized cognitive development	Adaptive learning platforms using ML to analyze learner data and tailor content; AI-generated virtual patient scenarios.	Addresses individual knowledge gaps; optimizes clinical reasoning skills through personalized learning pathways.	Intelligent tutoring systems that structure knowledge; virtual scenarios for clinical decision-making.	IV (26, 35, 41), I (25)
Zheng et al. (2023) (20), Turner et al. (2024) (35), Holmboe et al. (2023) (36)	Data-driven assessment and feedback	Automated skill assessment using video analysis and clinical reasoning text analysis.	Enables objective, quantifiable evaluation of competence; reduces subjectivity and provides timely, actionable feedback.	Automated scoring of procedural steps in simulations; ML evaluation of clinical notes.	III (20), IV (35, 36)
Su et al. (2023) (26), Cook et al. (2018) (31), Turner et al. (2024) (35), Holmboe et al. (2023) (36), Tammets et al. (2023) (41), Ara Shaikh et al. (2022) (87)	Educational management and optimization	AI analytics of aggregated student performance and resource utilization data.	Informs curriculum design and faculty deployment; enables proactive interventions and forecasts resource needs.	Data-driven insights for instructional adjustment; administrative decision-support for resource allocation.	IV (31, 35, 36, 41, 42), I (25)

Evidence level classification: Level I, randomized controlled trial or systematic review; Level II, prospective cohort study; Level III, retrospective study or pre-post study; Level IV, case series, proof-of-concept, or qualitative study; Level V, expert opinion or case report (7).

For educators, ML-driven analytics systems deliver real-time, data-driven insights into student learning processes. More importantly, research indicates that involving teachers in the collaborative design of theory-based dashboards and incorporating them into teacher training contributes to the development of teachers’ data interpretation skills and instructional decision-making abilities (41). By continuously analyzing performance and engagement metrics, data-driven intelligent tutoring platforms enable instructors to dynamically adjust instructional content and strategies, enabling timely interventions for struggling students and shifting education from a passive to an active, personalized paradigm (36, 42). Furthermore, VR enables the creation of patient scenarios and provides a safe environment for clinical skills training, while AI may further enhance the training efficacy of such VR platforms. Educators could potentially use AI-generated feedback to assist in evaluating trainee performance and providing targeted guidance, which holds potential value for tracking individual progress in medical education (43).

At the administrative level, AI empowers data-informed decision-making for resource optimization. By analyzing aggregated data on student performance, resource usage, and learning outcomes, ML-powered platforms can identify overarching trends and inefficiencies. This intelligence guides managers in making strategic decisions regarding curriculum design, faculty deployment, and the procurement of teaching materials (26, 31, 35, 39). The analytical capability of AI inherently supports forecasting resource utilizations, such as simulation equipment demand and pinpointing academic areas requiring intensified instructional support, thereby directing resources to maximize educational impact.

## 5 Challenges

Despite its promising applications, the integration of AI into anesthesiology education faces several significant challenges that must be acknowledged and addressed to ensure its responsible and effective implementation.

### 5.1 Overreliance and a “false sense of security”

A primary concern regarding immersive simulators is the risk of fostering overreliance on AI systems. Training with AI-guided devices, such as needle-guidance techniques, may create a “false sense of security,” leading to a state of “confident incompetence.” In this condition, clinicians may become overly dependent on technological aids, resulting in excessive confidence even when errors occur during unsupervised practice (44). This reliance carries clinical risks, particularly from the misidentification of anatomical structures by AI. Given this inherent limitation, technology-enhanced training should be positioned as a supplemental tool to, rather than a complete replacement for, traditional methods. Foundational experiences such as hands-on clinical practice in regional anesthesia and theoretical mastery of ultrasound anatomy must remain the cornerstone of education (45).

### 5.2 Incomplete skill transfer and educational gaps

The simulated environment, while valuable, cannot fully replicate all aspects of real-world clinical practice. As identified by Zheng et al., anesthesia interns using a virtual puncture platform reported that it replaced critical operational steps, such as towel placement, epidural catheter insertion, and application of auxiliary materials, which diminished their sense of participation and could lead to the neglect of these steps in actual practice. Furthermore, fundamental competencies like patient communication and observing patient responses cannot be adequately practiced in virtual settings (20). This incomplete transfer of skills highlights a significant gap in current AI-driven simulations. Meanwhile, deploying unvalidated AI models in education poses a clear pedagogical risk. Platforms designed for self-directed learning, if built on algorithms not rigorously assessed for educational efficacy, clinical accuracy, and safety, may reinforce incorrect techniques or poor clinical judgment. This concern underscores the requirement for thorough validation of such technologies before widespread adoption (32). The potential risks are analogous to those in clinical AI applications, where unvalidated models can lead to errors in patient care (34). This issue is compounded as more trainees turn to AI platforms for self-directed learning in lieu of faculty-led instruction. Although these tools provide flexibility, their educational value depends fundamentally on platform quality. Evidence suggests they are effective when embedded within a structured learning framework that includes expert guidance (46). In the absence of robust validation, AI platforms risk widening competency gaps among learners who rely on them without contextual feedback from educators.

### 5.3 Algorithmic bias and lack of humanistic reasoning

The performance and fairness of AI systems depend directly on their training data. If this data reflects societal or demographic biases, the underlying ML models may perpetuate or even amplify them, producing unfair or inaccurate results (47). A salient example is racial bias in pulse oximetry. As demonstrated by Sjoding et al., because the algorithms for these devices were primarily calibrated on lighter-skinned populations, they systematically overestimate arterial oxygen saturation in patients with darker skin. An AI model trained on such biased pulse oximetry data to predict hypoxemia would not only fail to correct this error but would perpetuate and potentially amplify it, leading to inaccurate risk assessments and clinical mismanagement for underrepresented racial groups (48). Furthermore, to support medical practice, current artificial intelligence lacks the necessary top-down associative reasoning and humanistic judgment, the latter comprising empathy, ethical reflection, and complex logic (19). In a study by Benzinger et al. (49) a questionnaire was distributed to German anesthesiologists to assess their attitudes toward an AI-driven preference prediction tool. The results suggest that although AI can improve prediction accuracy and reduce surrogate burdens, it also introduces concerns about bias, dehumanization, and interpretability. Moreover, automating such procedures risks losing major advantages offered by ethical case review.

### 5.4 Data privacy and security concerns

Beyond educational implications, the underlying infrastructure of AI introduces systemic challenges. AI educational tools often rely on large datasets containing sensitive patient information, raising serious privacy and security concerns. These include unauthorized data use in predictive analytics, loss of access control, and health information breaches that may significantly affect patients psychologically (50). Moreover, data retention policies for certain AI models lack transparency. Even after account deletion, data may not be fully erased, conflicting with regulations such as the Health Insurance Portability and Accountability Act in the United States and the General Data Protection Regulation (51). It is therefore essential to integrate privacy-preserving mechanisms, such as standardized data governance, encryption technologies, and strict access management into AI algorithm design, and this may help balance AI efficiency with the protection of patient privacy rights (52).

AI-driven educational tools, especially large language models, pose a formidable challenge to intellectual property and academic integrity through the generation of “original” content. First, by generating false or fabricated evidence, AI output can constitute a misrepresentation of source material, potentially infringing upon the moral rights of authors to the integrity of their original copyrighted works (53). More critically, the generative process operates as a non-transparent “black box.” Having absorbed vast amounts of copyrighted training materials such as textbooks and academic papers, the model produces outputs that cannot be traced to specific sources. This inability fundamentally circumvents academic citation norms, thus infringing upon the original authors’ rights of attribution and adaptation. Consequently, leading academic publishers such as JAMA and Nature explicitly prohibit listing AI as an author and mandate full disclosure of its use; these policies anchor accountability with human authors and establish a baseline for identifying potential infringement risks (54).

Furthermore, large language models have three inherent shortcomings in knowledge representation and fact generation. First, the issue of knowledge cutoff prevents them from autonomously acquiring new information after training is completed. Although this limitation can be mitigated through techniques such as retrieval-enhanced generation, the models themselves still lack the capacity for continuous learning. Second, the models lack metacognition of their own knowledge boundaries: they cannot accurately distinguish the known from the unknown and tend to produce plausible answers to all questions. This phenomenon of “hallucinations,” generating content that contradicts facts or context, is an intrinsic property of the model architecture, not a mere engineering oversight (55). Thirdly, an inherent tension exists between the training objectives of fluency and factual accuracy. To generate coherent text aligned with human preferences, the model sometimes sacrifices factual accuracy, producing so-called “white lies” (56).

It is worth noting that, from a cognitive perspective, large language models are essentially statistical language models, not cognitive entities grounded in real-world experience (57). Their training process relies on learning word co-occurrence patterns from vast textual data, yet they have never interacted directly with the physical world through sensory systems. This leaves the model without basic physical intuition: it can generate a fluent description of water being poured into a cup, yet fails to understand fluidity, container volume, or the operation of gravity. The model’s understanding of the world is built on second-hand knowledge derived from textual descriptions, forming a highly abstract symbolic mapping rather than a representation grounded in embodied experience (58). This language-centric form of knowledge results in a fundamental cognitive deficiency when tackling tasks that require physical common sense, sensory experience, or interaction with the real environment. Applications in the medical field, therefore, require the utmost caution (59).

### 5.5 Multidimensional costs and inequitable access

The implementation of AI in anesthesiology education requires substantial financial investment in hardware such as servers and VR equipment, custom software development, and ongoing maintenance, which together create a significant barrier to adoption and exacerbate educational inequality. The feasibility and scalability of AI technology vary dramatically across different economic contexts. Low- and middle-income countries face unique challenges due to limited infrastructure and high initial costs, while resource-rich environments in higher-income countries can develop a wider array of applications, thereby widening the global educational gap (60).

Beyond financial expenditures, the substantial environmental footprint of AI infrastructure constitutes a parallel and often hidden cost that further entrenches inequity. The lifecycle of AI systems, spanning from manufacturing energy-intensive hardware to training large models and operating data centers, consumes vast amounts of electricity and water while generating significant carbon emissions and electronic waste. For instance, training a single large model can emit hundreds of tons of CO₂, and data center cooling can place immense strain on local water supplies. Crucially, these environmental burdens are not borne equally: resource extraction and e-waste disproportionately impact marginalized communities, while the energy and water demands of AI infrastructure can export resource scarcity to other regions. This establishes a dual injustice: the benefits of educational technology are concentrated, while its planetary and health costs fall disproportionately on the most vulnerable populations. Therefore, ensuring equity in AI-driven education necessitates a dual approach: rigorous assessment of environmental impacts and a firm commitment to mitigating them through sustainable practices and responsible governance (61).

### 5.6 Resistance and the need for critical AI literacy

The advent of AI has engendered resistance among some educators and students. Educators may fear that AI will supplant their teaching roles, while students might perceive AI-driven instruction as less nuanced and formal, potentially weakening their independent critical thinking skills (20). Overcoming this resistance requires a multifaceted approach: encouraging participation in the development and refinement of AI tools to foster a sense of ownership, and providing comprehensive training for both educators and students. This training should cover not only the fundamentals of AI operation but also, and more importantly, the cultivation of critical thinking skills necessary to evaluate AI-generated content, such as that produced by large language models, and to use these tools judiciously as aids rather than replacements for human intellect (62).

### 5.7 Deeper ethical and regulatory considerations

The ethical discussion of artificial intelligence in medical education should extend beyond general concerns such as bias, privacy, and data security to include algorithm validation standards, regulatory governance, medico-legal accountability, and accreditation implications. These structural and policy-level issues are essential for ensuring that AI systems used in medical training are safe, reliable, and aligned with professional standards.

First, algorithm validation is a fundamental requirement for trustworthy AI systems in both clinical and educational settings. Unlike traditional educational technologies, AI-driven systems are data-dependent and may continuously evolve through model updates and new datasets. Therefore, validation should not be limited to technical performance metrics such as accuracy or area under the curve, but should also include external validation, clinical effectiveness evaluation, fairness assessment, transparency, explainability, and long-term monitoring after deployment. Policy analyses and AI governance reports emphasize that AI systems in healthcare should follow a lifecycle evaluation framework, including pre-deployment validation, real-world performance monitoring, and periodic reassessment to ensure safety and reliability over time (63–65). These recommendations highlight that algorithm validation should be considered a continuous process rather than a one-time technical assessment.

Second, regulatory frameworks play a critical role in governing the development and implementation of AI technologies in healthcare and medical education. Recent global reviews of artificial intelligence medical device regulation show that regulatory agencies such as the U. S. Food and Drug Administration, the European Union, China, Japan, and South Korea have adopted lifecycle-based regulatory models that emphasize risk-based classification, algorithm transparency, update control mechanisms, and post-market surveillance. However, regulatory standards remain heterogeneous across regions, and many AI systems still lack adequate clinical validation data and standardized evaluation frameworks. The lack of harmonized international regulatory standards presents challenges for the global implementation of AI technologies in medical education and clinical training, suggesting that future regulatory policies should address not only clinical AI applications but also AI-assisted educational technologies (66).

Third, the increasing use of AI in medical education and clinical decision-support training raises important medico-legal liability and accountability issues. When AI-assisted systems provide incorrect recommendations or training feedback that contributes to clinical errors, it becomes unclear whether responsibility lies with the clinician, educator, institution, or AI developer. Current legal frameworks often lack clear definitions regarding liability in AI-assisted decision-making environments (67). Policy analyses suggest that responsibility should be distributed across the entire AI lifecycle, including data collection, algorithm development, deployment, monitoring, and updating, in order to ensure patient safety and professional accountability (68). Establishing clear accountability frameworks is essential to promote responsible adoption of AI technologies in medical training and clinical decision support.

Finally, the integration of artificial intelligence into medical education also has implications for accreditation standards and competency frameworks. As AI becomes increasingly integrated into clinical practice, medical education accreditation bodies may need to incorporate AI literacy, digital ethics, and human-AI collaboration competencies into competency-based medical education frameworks (69, 70). Recent studies on AI curriculum development emphasize that structured AI education programs should include technical literacy, clinical application training, ethical governance, and interdisciplinary collaboration skills, and these competencies may eventually become part of accreditation and professional competency requirements (71).

Overall, the integration of artificial intelligence into medical education requires not only technological innovation but also robust validation standards, regulatory governance, legal accountability frameworks, and accreditation reforms. Without these structural safeguards, the rapid adoption of AI in education may introduce new risks related to safety, fairness, and professional responsibility. Therefore, future development of AI in medical education should be guided by multidisciplinary collaboration among educators, clinicians, policymakers, legal experts, and technology developers to ensure safe, ethical, and accountable implementation.

## 6 Future perspectives

### 6.1 Toward a human-centric, augmentative model

A key consideration emerging from this synthesis is that artificial intelligence may be most appropriately positioned as an augmentative tool within a human-centric model for anesthesiology education, rather than as a replacement for traditional teaching methods. This perspective is supported by the challenges identified in this review, including overreliance on automated feedback, potential deficits in non-technical skills, and AI’s inherent lack of humanistic reasoning. These limitations suggest that AI is unlikely to replace the nuanced clinical judgment, empathy, mentorship, and contextual decision-making provided by experienced educators and clinicians (19, 20, 49). Current evidence indicates that AI-driven simulations and ITS can support foundational training, procedural practice, and knowledge acquisition in controlled environments. However, most existing studies focus on simulation-based learning outcomes, skill acquisition, or learner satisfaction rather than long-term clinical performance or patient outcomes. Therefore, AI should be viewed as a supplementary educational tool that may support early skill development and knowledge reinforcement, while higher-order competencies such as clinical reasoning, communication, teamwork, and ethical decision-making continue to rely heavily on human instruction and mentorship. Gamification and AI-assisted interactive learning environments may improve learner engagement and provide opportunities to simulate complex patient management scenarios (72). Nevertheless, the educational effectiveness of these approaches remains dependent on instructional design, faculty involvement, and appropriate integration into existing curricula rather than the technology itself. Similarly, the increasing use of large language models such as ChatGPT in medical education illustrates the potential of AI to support learning and information retrieval, but concerns regarding accuracy, hallucinations, and overreliance require that such tools be used cautiously and under appropriate educational supervision (62). Overall, the available evidence suggests that the most realistic near-term role of artificial intelligence in anesthesiology education is likely to be augmentative rather than transformative. AI may support simulation training, feedback generation, and personalized learning pathways, but it is unlikely to replace human educators in areas requiring professional judgment, ethical reasoning, and mentorship. Future research should therefore focus not only on technological development but also on determining the appropriate balance between AI-assisted learning and traditional faculty-led education.

### 6.2 Overcoming implementation barriers: ethics, equity, and governance

Building upon the challenges outlined in Section 5, particularly algorithmic bias and data privacy, proactive strategies may be necessary to support responsible and equitable integration of artificial intelligence into anesthesiology education. The importance of these issues is highlighted by documented real-world harm associated with racial bias in pulse oximetry, an essential tool in anesthesiology practice (48). Such examples illustrate how technological systems can unintentionally perpetuate inequities if bias and data governance issues are not adequately addressed. Addressing these challenges may require both robust data governance policies and technical mitigation strategies, including deviation detection algorithms, fairness-aware modelling, and dataset diversification. Emerging approaches from other medical domains, such as enhanced chest X-ray image preprocessing and fairness-aware model training, suggest potential strategies for reducing bias in AI systems (73, 74). However, these approaches have not yet been extensively validated in anesthesiology-specific educational tools, and their effectiveness in educational settings remains uncertain. Further research is therefore needed to determine how bias mitigation strategies can be effectively implemented in AI-driven educational platforms. In parallel, data governance frameworks are likely to play an important role in ensuring responsible AI implementation. These frameworks may include strict access controls, transparent data usage policies, and privacy-preserving technologies such as federated learning, which allow model training without direct data sharing (75–77). Such approaches may help protect patient confidentiality while enabling collaborative model development across institutions. However, the implementation of these frameworks may vary depending on institutional resources, regulatory environments, and technical infrastructure. In addition to governance and technical challenges, economic and access-related barriers may influence the adoption of artificial intelligence in anesthesiology education. The development and implementation of AI technologies may be associated with high costs, specialized infrastructure, and technical expertise, which could contribute to unequal access between institutions with different resource levels. This raises concerns about a potential “digital divide” in medical education, where resource-rich institutions may benefit disproportionately from AI-enhanced training tools (60). Addressing this issue may require collaborative development models, open educational resources, and international partnerships to improve accessibility and equity. Finally, medico-legal liability remains an important but unresolved issue in the implementation of AI-assisted educational and decision-support systems. When AI-generated feedback or recommendations contribute to clinical errors or inappropriate training decisions, it may be unclear whether responsibility lies with the clinician, educator, institution, or technology developer. Clarifying legal responsibility and establishing accountability frameworks may therefore be necessary to support safe and responsible implementation of AI technologies in medical education and clinical training environments (78, 79). Overall, these ethical, governance, economic, and legal considerations suggest that the successful integration of artificial intelligence into anesthesiology education will depend not only on technological development, but also on governance frameworks, regulatory guidance, and institutional policies that ensure safe, equitable, and accountable use of AI technologies.

### 6.3 Evidence-based framework for AI curriculum integration in competency-based medical education

The integration of artificial intelligence into medical education may need to move beyond isolated pilot courses and optional workshops toward more structured curriculum integration aligned with competency-based medical education. Rather than treating artificial intelligence as a purely technical topic, educational programs may consider adopting an evidence-informed framework that integrates AI literacy, clinical application, ethical governance, and human-AI collaboration across different stages of medical training (80). Such an approach is consistent with the principles of competency-based medical education, which emphasize measurable competencies, longitudinal development, and outcome-based assessment rather than time-based training.

Recent systematic and narrative reviews indicate that effective integration of artificial intelligence into medical education may require structured curricula that combine technical knowledge, clinical reasoning, and ethical decision-making. AI education may therefore include core competencies such as data literacy, understanding algorithm limitations, interpretation of AI outputs, ethical use of AI tools, and collaborative decision-making between humans and AI systems. Importantly, artificial intelligence should not be viewed solely as a technological tool, but rather as a decision-support technology that requires critical evaluation, professional judgment, and ethical responsibility from learners and clinicians.

Within the competency-based medical education framework, artificial intelligence may also have the potential to support competency assessment, personalized learning, and longitudinal performance tracking. AI-based educational systems could provide automated feedback, simulation-based training, adaptive learning pathways, and more objective assessment of procedural and communication skills. These technologies may help educators track learner progression across multiple competency domains, including clinical knowledge, procedural skills, communication, professionalism, and clinical decision-making (81). However, the reliability, validity, and educational impact of AI-assisted assessment tools remain areas that require further validation and comparative educational research. An evidence-based curriculum integration framework for artificial intelligence in medical education may be conceptualized as a staged model across the medical training continuum. At the undergraduate level, the curriculum should focus on foundational AI literacy, including basic concepts of machine learning, data bias, and ethical considerations. During clinical training and residency, the focus may shift toward clinical applications of AI, including decision-support tools, simulation training, and interpretation of AI-assisted diagnostic outputs (82). At the continuing professional development level, education may emphasize advanced topics such as AI governance, regulatory frameworks, medico-legal responsibility, and interdisciplinary collaboration with engineers and data scientists (83). This staged integration model may allow AI education to develop progressively alongside clinical competencies, although empirical evidence supporting specific implementation models remains limited.

In addition to curriculum content, institutional support and faculty development are likely to be essential for successful AI integration. Many educators currently lack formal training in artificial intelligence, which may limit the effective implementation of AI-based educational tools. Faculty development programs, interdisciplinary collaboration, and institutional governance structures may be necessary to support curriculum integration and ensure responsible AI use in education. Furthermore, accreditation bodies and medical education regulators may in the future consider incorporating artificial intelligence competencies into competency frameworks and accreditation standards to ensure that physicians are prepared for AI-assisted clinical practice (84).

Overall, an evidence-based framework for artificial intelligence curriculum integration may include four key components: foundational AI literacy, clinical application training, ethical and legal education, and competency-based assessment supported by AI tools. However, the optimal structure, timing, and educational effectiveness of such frameworks remain areas for future research. Artificial intelligence is therefore likely to function as a complementary educational tool within competency-based medical education rather than as a replacement for traditional teaching and mentorship.

### 6.4 Future directions for research and development

Current evidence, while promising, also highlights several important gaps that warrant further investigation. Future research may need to focus not only on demonstrating skill acquisition and learner satisfaction, but also on evaluating the impact of AI-enhanced training on patient outcomes and clinical performance, corresponding to higher levels of the Kirkpatrick evaluation framework. A key unanswered question is whether AI-assisted training ultimately translates into improved patient safety and care quality, which will likely require longitudinal studies and real-world clinical outcome data (27).

In addition, further research is needed to determine how and when artificial intelligence should be integrated into existing curricula. This includes identifying the most effective ways to embed simulation-based AI training within traditional educational structures and clarifying its potential role in mitigating the decline of clinical skills over time (26). At present, the optimal timing, duration, and intensity of AI-assisted training remain unclear and may vary depending on training level, specialty, and institutional resources.

Two critical domains, directly extending from the challenges identified in Section 5, also demand further investigation. The first involves the transition from “black box” AI assessment systems toward explainable and interpretable systems that provide meaningful feedback to support learning rather than simply generating performance scores. The second involves the continued development of methods to mitigate algorithmic bias and protect data privacy, both of which remain major barriers to wider implementation of AI technologies in medical education.

AI educational platforms should be rigorously evaluated before widespread implementation, particularly those designed for self-directed learning. These evaluations may need to assess not only immediate skill acquisition, but also long-term retention, transfer of skills to clinical practice, and the development of clinically appropriate decision-making behaviors. Comparative effectiveness research comparing AI-augmented self-directed learning with traditional faculty-led training may also be valuable in determining the contexts in which each approach is most appropriate and effective (85). Such research would help clarify not only whether AI-assisted educational tools are effective, but also how, when, and for whom they are most beneficial.

Overall, future research should aim to establish stronger evidence regarding the educational effectiveness, clinical impact, and appropriate implementation strategies for artificial intelligence in anesthesiology education. Until such evidence is available, artificial intelligence should be considered a promising but still evolving educational tool rather than a fully established solution.

## 7 Limitations

Several methodological limitations of this review should be acknowledged. First, despite employing a structured search strategy, we cannot exclude the possibility of selection bias. We limited the search to three databases, namely PubMed, Web of Science, and ERIC, and although this combination was chosen to cover both clinical and educational literatures, relevant studies indexed in other databases may have been missed. Additionally, including only English-language articles may introduce language bias and limit the generalizability of findings to non-English-speaking contexts.

Second, as this is a narrative rather than a systematic review, we did not perform a formal risk-of-bias assessment or publication bias analysis. The evidence level classification presented in Table 2 reflects study design rather than a comprehensive appraisal of methodological quality. Consequently, the synthesis should be interpreted as a qualitative summary of the current landscape, not as a quantitatively pooled estimate of effect.

Third, the rapid evolution of AI technologies outpaces any static review. Although we aimed to capture the most recent literature with a final search update in March 2026, new applications and studies may have emerged since then.

## 8 Conclusion

AI integration marks a paradigm shift in anesthesiology education with enhanced efficiency, personalization, and efficacy. AI technologies ranging from intelligent simulators to adaptive learning platforms are demonstrably transforming how psychomotor skills, clinical reasoning, and procedural fluency are acquired and evaluated. This transition empowers a more dynamic and engaging learning environment, potentially bridging critical gaps in hands-on experience and theoretical knowledge.

However, the path forward is not merely technological, but also fundamentally human-centric. The promise of AI must be balanced against the significant challenges discussed in Section 5, underscoring that AI’s optimal role is that of an augmentative tool, designed to empower rather than replace the irreplaceable mentorship, nuanced judgment, and empathetic communication of clinical educators. The goal is to cultivate a synergistic model where AI handles scalable tasks of knowledge delivery and repetitive practice, thereby freeing educators to focus on fostering higher-order cognitive skills and professional virtues.

To secure this future, a concerted, multi-stakeholder effort is required. Educators and institutions must develop critical AI literacy and integrate these tools under frameworks that prioritize ethical governance, equitable access, and continuous curriculum evaluation. Researchers must advance the field by pursuing longitudinal studies that link AI-enhanced training to tangible improvements in patient outcomes (Kirkpatrick Level 4), while also developing explainable and fair AI systems. Policymakers and professional societies are tasked with creating clear guidelines for accountability and data security to foster trust and responsible implementation.

Ultimately, the aim is not to produce a generation of technicians dependent on algorithms, but to equip a new cadre of anesthesiologists who are discerning experts, capable of leveraging technology as a powerful ally in their unwavering commitment to delivering safe, high-quality, and compassionate patient care. By navigating the current challenges with foresight and responsibility, the anesthesiology community can harness the full potential of AI to shape the future of medical education and clinical practice.
